# Effectiveness of a Mobile Wellness Program for Nurses with Rotating Shifts during COVID-19 Pandemic: A Pilot Cluster-Randomized Trial

**DOI:** 10.3390/ijerph19021014

**Published:** 2022-01-17

**Authors:** Yeongmi Ha, Sang-Ho Lee, Dong-Ha Lee, Young-Hun Kang, Woonjoo Choi, Jinung An

**Affiliations:** 1 School of Nursing, Institute of Health Sciences, Gyeongsang National University, Jinju 52727, Korea; yha@gnu.ac.kr; 2 Convergence Research Advanced Centre for Olfaction, DGIST, Daegu 42988, Korea; comman35@dgist.ac.kr; 3 Division of Intelligent Robot, DGIST, Daegu 42988, Korea; dhlee@dgist.ac.kr; 4 BIOSFIT, Jinju 52650, Korea; lohas_co@naver.com; 5 Department of Nursing, Gyeongsang National University Hospital, Jinju 52727, Korea; cwjlove@gnuh.co.kr; 6 Department of Interdisciplinary Engineering, DGIST, Daegu 42988, Korea

**Keywords:** nurses, mobile health, self-efficacy, wellness, physical activity, sleep quality

## Abstract

Nurses with rotating shifts, including night shifts, have suffered from low physical activity during the COVID-19 pandemic and lower sleep quality due to the disruption of their circadian rhythm. This study aimed to develop and examine the effectiveness of a mobile wellness program on daily steps, sleep quality, exercise self-efficacy, intrinsic motivation for exercise, self-rated fatigue, and wellness. A cluster randomized controlled trial design was used to examine the effectiveness of the mobile wellness program for nurses with rotating shifts. Sixty nurses from one university hospital participated and were allocated to an intervention group and a control group. The intervention group received a 12-week mobile wellness program to improve their physical activity and sleep quality, and the control group was only given a Fitbit to self-monitor their health behaviors. There were significant differences between the two groups in daily steps (*p* = 0.000), three components (subjective sleep quality, sleep disturbance, daytime dysfunction) of the PSQI, exercise self-efficacy, intrinsic motivation for exercise, and wellness. In conclusion, this study provides meaningful information that the mobile wellness program using Fitbit, online exercise using Zoom, online health coaching on a Korean mobile platform, and motivational text messages effectively promoted physical activity and sleep quality for nurses with rotating shifts during the COVID-19 pandemic.

## 1. Introduction

The exposure of nursing staff to shift work is inevitable because hospital nurses need to provide continuous patient care around the clock. According to the Korean Nurse Association [1], registered nurses in Korean hospitals work six or more night shifts per month. It is well known that shift work disrupts circadian rhythms, which in turn leads to difficulties with falling asleep due to the misalignment of sleep with the circadian rhythm [2]. Previous studies have shown that shift nurses have lower sleep quality than non-shift nurses because shift work increases the risk of sleep disturbance [3,4]. In particular, the sleep quality of nurses due to night shifts could increase the risk of cardiovascular disease caused by sympathetic nervous system activation, emotional dysregulation, fatigue, and daytime sleepiness [5].

During the COVID-19 pandemic, nurses are subject to challenging occupational demands, including long working hours, physically demanding workloads, and irregularly rotating shifts. Such poor working environments have been associated with nurses’ health-risk behaviors, including emotional eating, high-energy snacking, and low levels of physical activity, which result in obesity and cardiovascular diseases [6]. Although nursing seems to include frequent walking bouts, nurses appear not to be meeting the current physical activity guidelines of 150 min per weekend, demonstrating low physical activity [7].

Regular physical activity and good sleep play an essential role in preventing negative occupational and health consequences [8]. Physical activity is a key representative non-pharmacological factor that positively affects sleep quality [9]. Systematic reviews of adults with sleep problems and individuals across their lifespan have shown that both long-term and acute bouts of physical activity positively affect sleep [10,11]. A few studies on the relationships between physical activity and sleep for shift workers have demonstrated that physical activity programs for shift workers improved their quality of sleep, including their sleep efficiency and sleep latency [12]. Nurses are unlikely to participate in physical activity programs provided by institutions or public health centers due to poor work environments with irregular shifts, night shifts, and long working hours. Therefore, the customized interventions for nurses with rotating shifts are required to improve the physical activity and sleep quality provided in everyday lives because nurses working rotating shifts or night shifts have poorer health-promoting behaviors and fewer opportunities for exercising than non-shift workers [13].

Mobile health technology, including wearable activity trackers and smartwatches, might effectively engage shift workers in wellness programs [12]. Since the COVID-19 pandemic has caused us to adjust to life indoors, efforts to reduce the spread of the virus have forced people to adapt to online exercise using Zoom, Instagram, and YouTube [14]. As various wearable tracking devices and smartwatches have been developed, which can positively influence health behaviors, such as increasing physical activity, research on mobile wellness programs to promote physical activity has been actively conducted [15]. Wearable tracking devices have been demonstrated to help people change their health behaviors by providing daily steps and sleep data, allowing them to self-monitor their health behavior [16]. To date, however, studies on mobile wellness programs for promoting physical activity and sleep quality among nurses are lacking even though a large number of studies have examined the effectiveness of mobile wellness programs targeting adults of various ages. If wearable tracking devices and online health activities could be linked to a mobile platform, compliance with health-promoting behaviors may be improved. Therefore, this study aimed to develop a mobile wellness program to promote physical activity and sleep quality using Fitbit, online exercise, health coaching through a Korean mobile platform, and motivational text messages. The purpose of this study was to examine the effectiveness of the program on daily steps, sleep quality, exercise self-efficacy, intrinsic motivation for exercise, self-rated fatigue, and wellness.

## 2. Materials and Methods

### 2.1. Study Design

A cluster randomized controlled trial design was used to examine the effectiveness of the mobile wellness program for nurses with rotating shifts to promote physical activity and sleep quality.

### 2.2. Participants and Randomization

The target population for this study was registered nurses working 8-h rotating shifts in Korea. The participants were 60 nurses working in a university hospital employing approximately 10,000 nurses. The inclusion criteria of participants were age between 19 and 60 years, working in surgical or medical wards for over four nights per month, not participating in any exercise programs within the past six months, and having a Fitbit-compatible smartphone for downloading the Fitbit app. Exclusion criteria were contraindications for exercise, such as chest pain, cardiovascular diseases, and musculoskeletal disorders. Participants could be excluded if they were diagnosed with sleep disorders. 

The cluster-randomized controlled trial was adopted because this design might eliminate the risk of treatment contamination. The randomization was implemented using computer-generated random numbers with a 1:1 allocation using a random sequence of blocks of 5 nurses in each ward instead of individual nurses. The intervention group participated in the mobile wellness program to improve physical activity and good sleep quality, while the control group was only given a Fitbit. 

The sample size estimation was used by the G*Power 3.1.9.2 program. The criteria for independent *t*-test were as follows: statistical power (1-β) of 0.80, significance level (α) of 0.05, effect size (d) of 0.50. It was indicated that a total of 54 participants (27 people per group) were required. Allowing for 10% attrition, we recruited 60 participants for the study, with 30 in the intervention group and 30 in the control group. As no one in the intervention group and three nurses in the control group withdrew from the program, a total of 57 datasets were analyzed. 

### 2.3. Measurements

#### 2.3.1. Daily Steps

Participants were asked to wear a Fitbit Versa 24 h a day to measure their daily steps. Daily steps were objectively measured using a triaxial accelerometer (Fitbit Versa; Fitbit Inc., San Francisco, CA, USA), a valid and reliable measure of physical activity in adults [17]. The data were automatically uploaded to the Fitbit app on the participant’s smartphone and simultaneously uploaded on a data-management platform called DGIST Wellness, which allows for the collection of data every minute. We represented daily steps as a mean value of the total walking steps, calculated as the sum of daily steps divided by the number of days. In addition, 5000~9999 steps/day was classified as low to somewhat active, and ≥10,000 steps/day was classified as active to highly active based on previous studies [18]. 

#### 2.3.2. Sleep Quality 

The Korean version of the Pittsburgh Sleep Quality Index (PSQI-K) was used to assess sleep quality [19], which was a self-rated questionnaire composed of 19 items subsequently classified into the following seven components: subjective sleep quality, sleep latency, sleep duration, habitual sleep efficiency, sleep disturbances, use of sleeping medication, and daytime dysfunction during the past month. Each component was weighted from 0 (no difficulty) to 3 (severe difficulty), generating one global score ranging from 0 to 21. Poor sleep quality was indicated by a total score of 8.5 or greater, while good sleep quality was indicated by a score of less than 8.5 in Korea [19]. The Cronbach’s α in this study was 0.88 for sleep quality.

#### 2.3.3. Exercise Self-Efficacy 

The exercise self-efficacy assesses confidence in the ability to exercise in high-risk situations [20]. The Korean version of the Self-efficacy for Exercise Scale (SEE-K) consists of 10 items, and higher scores represent higher self-efficacy for exercise. The responses ranged from 1 (not at all true) to 4 (always true). The total scores ranged from 8 to 40; higher scores indicate greater exercise self-efficacy. The Cronbach’s alpha for this study was 0.95 for exercise self-efficacy. 

#### 2.3.4. Intrinsic Motivation for Exercise 

The Behavioral Regulation in Exercise Questionnaire-2 (BREQ-2) translated into Korean was used in this study to assess intrinsic motivation for exercise [21]. This instrument comprised four components, including external (4 items, e.g., “I exercise because other people say I should”), introjected (3 items, e.g., “I feel guilty when I do not exercise”), identified (3 items, e.g., “I value the benefits of exercise”), and intrinsic (4 items, e.g., “I exercise because it is fun”) regulations. In addition, four amotivation items from the initial item pool were included (“I do not see why I should have to exercise”, “I cannot see why I should bother exercising”, “I do not see the point in exercising”, and “I think that exercising is a waste of time”). Due to an error, one item was omitted from the original BREQ identified subscale (“I get restless if I do not exercise regularly”). Responses were scored on a 5-point Likert scale ranging from 0 (not true for me) to 4 (very true for me). The Cronbach’s α was 0.86 in this study.

#### 2.3.5. Self-Rated Fatigue

The Multidimensional Fatigue Scale [22] translated into Korean was used to measure people’s fatigue. A 19-item self-report instrument consisted of three components (6 items on daily life dysfunction, 8 items on general fatigue, and 5 items on contextual fatigue). Responses were made on a 6-point Likert scale, where the higher the score is, the more severe the fatigue is. The Cronbach’s α was 0.91 in this study.

#### 2.3.6. Wellness

The World Health Organization’s Health Promotion Glossary described wellness as the optimal state of health and the realization of the fullest potential of a physically, socially, psychologically, economically, and spiritually and the fulfillment of a person’s role expectation [23]. The wellness index for Korean workers [23] was used to measure workers’ wellness. An 18-item self-report instrument comprised the following five components: physical wellness (4 items), emotional wellness (5 items), social wellness (3 items), intellectual wellness (3 items), and occupational wellness (3 items). Responses were scored on a 5-point Likert scale ranging from 0 (not true for me) to 4 (very true for me), where the higher the score is, the better the wellness is. The Cronbach’s α was 0.92 in this study. 

### 2.4. A Mobile Wellness Program for Shift Nurses

A mobile wellness program for nurses with rotating shifts to promote physical activity and sleep quality was designed for 12 weeks, where Fitbit, online exercise using Zoom, online health coaching through Kakao Talk as a Korean mobile platform, and motivational text messages to set up the long-term goal and short-term goals were provided to the intervention group. 

The Fitbit allowed nurses to self-monitor real-time activity and sleep patterns. In week 1 of the mobile wellness program, the objective was to recognize their physical activity level by assessing the frequency, intensity, and duration of physical activity. The nurses walking 5000~9999 steps a day aimed to gradually increase to 10,000 steps a day during the 1st~6th week of the mobile wellness program, and the goal of nurses walking over 10,000 steps a day was to maintain their activity level. In the 7th~12th week of the mobile wellness intervention, the goal was to add 1000 extra steps a day every two weeks and exercise intensity at 50~60% of the target heart rate.

Live, online group exercise was delivered through Zoom to stay active during the COVID-19 pandemic. The online exercise was designed to allow participants to enjoy live interactions with the instructors and personal feedback. Online exercise was held for an hour every Tuesday and Thursday morning. Furthermore, 30-min strengthening exercise videos were uploaded on Kakao Talk, which is a Korean mobile platform that participants could use to freely watch the videos anytime and anywhere. 

During online health coaching, the participants were encouraged to set long-term goals through the 12-week mobile wellness program and short-term goals for each week. Weekly online health coaching was provided, including checking whether daily steps were reached based on their short-term goals in the previous week, the dosage of aerobic exercise about the frequency, intensity, and duration of exercise, offering problem-solving strategies to improve exercise self-efficacy and teaching ways to increase the amount of physical activity early in the program. Online health coaching included monitoring participants’ target heart rate, checking the target heart rate on a Fitbit, and the meaning of 50~60% of the target heart rate in the second half of the mobile wellness program. The participants were informed that the mobile platform helped users interact with other users and research teams to discuss goal attainment and overcome barriers to physical activity and sleep problems.

Based on a daily step account through Fitbit and the DGIST Wellness platform, personalized motivational text messages were sent to the participants through Kakao Talks, such as “Monday is the day to set your goal for daily walking steps! Your target walking step is (each participant’s walking step in this week) steps.” The participants in the control group received a Fitbit to self-monitor their activity and sleep patterns only.

### 2.5. Data Collection

The data collector and participants were blinded to minimize the risk of biased outcomes. Data were collected during the COVID-19 pandemic from March to June 2021. A data collector with ten years of nursing experience as a nurse was hired to conduct the survey; they were blinded to participant allocation. Instructions for filling in the questionnaire were given to the data collector. Data were measured immediately before the intervention as pretest and after the completion of the intervention as post-test.

### 2.6. Ethical Considerations

Ethical approval was obtained by the institutional review board of a university hospital (IRB No: GNUH2020-12-015, 12 January 2021), and then, the purpose of the study and its procedures were explained to the participants. All participants were informed that their participation was voluntary, and they were free to withdraw from the research at any time without any loss of benefits. Each questionnaire was distributed to participants who provided voluntary informed consent to participate in this study.

### 2.7. Statistical Analyses

Data were analyzed using the SAS 9.4 (SAS Institute, Cary, NC, USA) program. All variables were checked for outliers. Normality was checked using the Shapiro–Wilk test, and all variables satisfied the assumption of normality. A homogeneity test on participants’ general characteristics was performed using an independent *t*-test or chi-square with Fisher’s exact test. Moreover, an independent *t*-test was used to examine the homogeneity of daily steps, sleep quality, exercise self-efficacy, intrinsic motivation for exercise, self-rated fatigue, and wellness between the intervention and control group before the program. Finally, the effectiveness of a mobile wellness program on daily steps were examined at 7 and 12 weeks using the repeated measures ANOVA. The effectiveness of the program on sleep quality, exercise self-efficacy, intrinsic motivation for exercise, self-rated fatigue, and wellness at 12 weeks was tested using an independent *t*-test.

## 3. Results

### 3.1. Homogeneity of General and Job-Related Characteristics in Participants

There were no statistically significant differences in the general and job-related characteristics between the intervention and control groups (Table 1). There were 30 participants in the intervention group and 27 participants in the control group. 

### 3.2. Homogeneity of Behavioral Characteristics in Participants

Table 2 shows no statistically significant differences between the two groups on daily steps, sleep quality (PSQI total score), the seven components of the PSQI, exercise self-efficacy, intrinsic motivation for exercise, self-rated fatigue, and wellness (Table 2).

### 3.3. Effectiveness of a Mobile Wellness Program on Daily Steps

There were statistically significant differences between the two groups on daily steps at the 7th week (t = 2.74, *p* = 0.008) and at the 12th week (t = 3.52, *p* = 0.000). The intervention group reported statistically increased daily steps relative to the control group in both the 5000~9999 steps/day (t = 2.43, *p* = 0.022) and over 10,000 steps/day ranges at the 12th week (t = 2.91, *p* = 0.007). There were statistically significant interactions between the groups and times for daily steps (F = 14.41, *p* < 0.001), 5000~9999 steps (F = 6.58, *p* = 0.002), and over 10,000 steps (F = 8.59, *p* = 0.001) although there was no significant difference across time points (Table 3).

### 3.4. Effectiveness of a Mobile Wellness Program

There were statistically significant differences between the two groups on the subjective sleep quality component of the PSQI (t = −2.29, *p* = 0.025), sleep disturbance component of the PSQI (t = −2.00, *p* = 0.049), daytime dysfunction component of the PSQI (t = −2.04, *p* = 0.045), exercise self-efficacy (t = 2.57, *p* = 0.012), intrinsic motivation for exercise (t = 2.78, *p* = 0.007), and wellness (t = 4.14, *p* = 0.000). However, there was no significant difference between two groups on sleep quality (PSQI total score) (t = −1.53, *p* = 0.161) and self-rated fatigue (t = −1.06, *p* = 0.295) (Table 4).

## 4. Discussion

This study provides meaningful knowledge that a mobile wellness program for nurses with rotating shifts during the COVID-19 pandemic effectively promoted physical activity and sleep quality. The mean age of the nurses participating in this study was 27.3 years old, and over 85% of them were young unmarried female workers. Rotating shifts in nursing are common to ensure continuous patient care; this scheduling system could negatively affect nurses’ health, quality of life, and nursing performance [6]. In particular, the adverse effects of night shift work due to the disruption of the circadian rhythm could be worsened in young female workers [2,24]. In addition, even though there was a decrease in the level of outdoor/indoor physical activity due to the COVID-19 pandemic, it was difficult to find a mobile physical activity program for nurses suffering from heavy workload caused by COVID-19. Therefore, implementing this mobile wellness program for shift nurses to promote physical activity and sleep quality using a Fitbit, online exercise, and online health coaching was important during COVID-19 pandemic.

The daily steps measured by Fitbit have shown a significant difference between the intervention group and the control group, where the intervention group significantly increased their daily steps by 1705, whereas the control group decreased their daily steps by 1305. This is in line with the findings of previous studies on mobile programs using activity trackers, such as Fitbit [25,26,27]. Systematic reviews on the step-based recommendations of physical activity guidelines have shown that 10,000 steps/day is appropriate for the health promotion of adults [28]. It is a meaningful result that shift nurses in the intervention group have reached an average of 11,000 steps per day after the 12-week mobile wellness program despite a lack of opportunities for physical activities due to their demanding workload and COVID-19.

An encouraging finding is that both the nurses walking 5000~9999 steps and those walking over 10,000 steps in the intervention group significantly increased their number of daily steps by more than 1617~1794 steps/day, while nurses in the control group decreased their daily steps by 791~1858 steps/day. What is noteworthy about this study is that the daily steps of the intervention group walking 5000~9999 steps increased significantly by a large margin, and those nurses achieved 10,000 steps/day for the target of healthy adults [28]. These improvements may be attributable to the detailed motivational strategies, such as tailoring text messaging, online health coaching through Kakao Talk as a mobile platform, and online exercise by a professional health trainer. It is also possible that our program supports various strategies for achieving goals, such as feedback messaging and rewards for achieving 10,000 steps/day.

The changes in sleep quality in this study were as not great in magnitude as was anticipated. Even though group differences in sleep quality (PSQI total score) were not statistically significant at 12 weeks, significant changes in some of the components of the PSQI were exhibited. In particular, significant improvements in subjective sleep quality, sleep disturbance, and daytime dysfunction were observed in the intervention group. The improvement in subjective sleep quality and sleep disturbance in the intervention group were likely to result from adherence to sleep hygiene activities. On the other hand, nonsignificant changes may be explained by the fact that the short study duration was not long enough to leverage a larger improvement of sleep quality. Considering the benefits of exercise and positive effects on sleep quality [29,30], exercise should be encouraged in nurses suffering from the adverse effects of rotating shifts.

Exercise self-efficacy and intrinsic motivation for exercise in the intervention group significantly increased after the mobile wellness program, whereas these slightly decreased in the control group, with a statistically significant difference between the two groups. These results are in line with previous studies that have demonstrated significant increases in exercise self-efficacy [25,26,27] and intrinsic motivation for exercise [21] after mobile programs using Fitbit. It is well-known that exercise self-efficacy is the most powerful predictor of exercising [25,27], and the assumption that efficacy and intrinsic motivation are closely related is established from motivation theories [31]. According to the motivation theories, feelings of efficacy should be experienced as intrinsically rewarding, and a higher level of perceived competence entails higher intrinsic motivation levels [31]. The reason for increases in exercise self-efficacy and intrinsic motivation in this study is that the mobile wellness program helped nurses experience successful exercising through individualized short-term and long-term goals for daily steps.

The nurses’ wellness in the intervention group significantly increased after participating in the mobile wellness program, while there was no significant difference in the control group, showing a statistically significant difference in wellness between the two groups. This finding is consistent with previous studies that nurses with a high level of physical activity reported improved health and well-being versus those who had a low-level of physical activity [32]. Regular exercise improves physical health and improves wellness and quality of life through mental and emotional well-being. The significant increase in the nurses’ physical, mental, and social wellness in this study could be explained by increased physical activity, various education through individual counseling, and customized text messages for 12 weeks, and this satisfaction seems to have improved the workers’ physical, mental, emotional, cognitive, and professional wellness. Wellness is known to play an important role in improving the well-being of individual workers, reducing presentation, absenteeism, and corporate productivity [33]; therefore, it is believed that mobile wellness programs for shift nurses will be useful in the future.

Self-rated fatigue slightly decreased in the intervention and control groups, but the between-group difference was not statistically significant in this study. Fatigue in nurses is widely prevalent and consequential because nurses are inevitably exposed to various personal and environmental characteristics leading to fatigue, such as long working hours and successive night shifts [34,35,36]. According to previous studies, regular exercise could help mitigate fatigue and help restore physical/emotional function more rapidly by augmenting recovery [34,35,36]. For example, nurses engaging in regular exercise reported lower fatigue levels and better recovery outcomes compared to those who did not regularly exercise [35]. Unfortunately, despite numerous pieces of evidence of significant relationships between fatigue resilience and regular exercise, our findings did not support such an association. Our findings should require further verification by implementing rigorous intervention with randomized controlled trials (RCT) and large-scale RCTs. Moreover, it is necessary to explore effective exercise programs, such as strength and endurance training, and the optimal intensity and volume of exercise for maximizing fatigue resilience.

To the authors’ knowledge, this study is the first trial to verify the effectiveness of the mobile wellness program for nurses suffering from the disruption of circadian rhythms and a low level of exercise during the COVID-19 pandemic. Although the mobile wellness program can be assumed to be effective to nurses, there are some considerations in this study. First, this study comprised a small sample of a relatively homogenous group of nurses from one university hospital, and the results may not be generalizable to nurses with rotating shifts. Second, all the participants in this study are female nurses in a university hospital employing approximately 10,000 nurses, and thus, it could not be generalized to male nurses or female nurses working in small/medium-sized hospitals. Third, implementation outcomes, such as the satisfaction with the intervention, barriers or enablers of Fitbit and mobile platform use, and likelihood of continued usage of online exercise and online health coaching, were not assessed in this study. Future research should evaluate implementation outcomes because the identification of such measures is critical to the dissemination, implementation, and generalizability of the mobile wellness program.

## Figures and Tables

**Table 1:** Homogeneity of General and Job-related Characteristics.

Characteristics	Categories	IG (*n* = 30) *n* (%) or M ± SD	CG (*n* = 27) *n* (%) or M ± SD	t or χ^2^ (*p*) ^†^
Sex	Female	30 (100.0)	27 (100.0)	-
Age	≤30	27 (90.0)	22 (81.5)	0.85 (0.355)
	>30	3 (10.0)	5 (18.5)	
	Mean ± SD	27.63 ± 3.05	27.03 ± 3.69	0.48 (0.630)
Marital status	Unmarried	26 (86.7)	23 (85.2)	0.02 (0.872)
	Married	4 (13.3)	4 (14.8)	
Educational level	College	5 (16.7)	6 (22.2)	0.28 (0.595)
	More than University	25 (83.3)	21 (77.8)	
Subjective economic status	Below Middle Low	7 (23.3)	2 (7.4)	2.71 (0.099)
	Middle	23 (76.7)	25 (92.6)	
Subjective health status	Poor	8 (26.6)	5 (18.5)	3.04 (0.385)
	Moderate	17 (56.7)	17 (63.0)	
	Healthy	5 (16.7)	5 (18.5)	
Clinical experience in	≤10	28 (93.3)	26 (96.3)	0.25 (0.616)
nursing (years)	>10	2 (6.7)	1 (3.7)	
	Mean ± SD	4.40 ± 3.87	4.40 ± 3.87	0.43 (0.668)
Position	Staff nurse	30 (100.0)	27 (100.0)	-
Night shifts per month	≤6	17 (56.7)	19 (70.4)	1.14 (0.284)
(days)	>6	13 (43.3)	8 (29.6)	
	Mean ± SD	6.28 ± 0.53	5.98 ± 1.03	1.76 (0.084)
A pattern of rotating shifts	Irregular	24 (80.0)	18 (66.7)	1.39 (0.497)
	Regular	6 (20.0)	9 (33.3)	

Abbreviation: CG, control group; IG, intervention group; SD, standard deviation. ^†^ Fisher’s exact test.

**Table 2:** Homogeneity of Behavioral Characteristics.

Variables	Range	IG (*n* = 30) M ± SD	CG (*n* = 27) M ± SD	t (*p*)
Daily steps	-	9950.2 ± 1830.4	10,204.8 ± 2672.9	−0.42 (0.673)
Sleep quality (PSQI total score)	0–21	9.20 ± 3.11	8.74 ± 3.18	0.55 (0.584)
Subjective sleep quality	0–3	1.63 ± 0.49	1.40 ± 0.63	1.51 (0.136)
Sleep latency	0–3	2.13 ± 0.86	2.14 ± 0.71	−0.07 (0.944)
Sleep duration	0–3	1.13 ± 1.10	1.03 ± 0.97	0.35 (0.730)
Habitual sleep efficiency	0–3	1.00 ± 1.23	1.07 ± 1.20	−0.23 (0.819)
Sleep disturbance	0–3	1.23 ± 0.50	1.18 ± 0.48	0.37 (0.714)
Use of sleeping medication	0–3	0.40 ± 1.67	0.48 ± 1.78	−0.18 (0.859)
Daytime dysfunction	0–3	1.66 ± 0.71	1.40 ± 0.79	1.30 (0.199)
Exercise self-efficacy	0–10	2.67 ± 1.48	3.37 ± 1.88	−1.58 (0.120)
Intrinsic motivation for exercise	1–5	3.26 ± 0.36	3.35 ± 0.65	−0.71 (0.482)
Self-rated fatigue	19–133	92.53 ± 14.61	94.81 ± 14.67	−0.59 (0.559)
Wellness	1–5	2.98 ± 0.47	3.18 ± 0.42	−1.68 (0.099)

Abbreviation: CG, control group; IG, intervention group; PSQI, Pittsburgh Sleep Quality Index; SD, standard deviation.

**Table 3:** Effectiveness of a Mobile Wellness Program on Daily Steps.

Variables		IG (*n* = 30) M ± SD	CG (*n* = 27) M ± SD	t (*p*)	Source	F(*p*)
Daily steps	1st week	9950.2 ± 1830.4	10,204.8 ± 2672.9	−0.42 (0.673)	Group	5.76 (0.019)
	7th week	10,939.7 ± 2206.1	9015.0 ± 3061.5	2.74 (0.008)	Time	0.56 (0.574)
	12th week	11,650.0 ± 2843.4	8899.7 ± 3058.2	3.52 (0.000)	Group × Time	14.41 (<0.001)
5000~9999 steps ^†^	1st week	8554.9 ± 1322.0	8322.4 ± 1095.2	0.51 (0.616)	Group	7.54 (0.011)
	7th week	9980.9 ± 2023.1	7285.6 ± 1897.6	3.64 (0.001)	Time	0.79 (0.459)
	12th week	10,348.3 ± 2939.9	7531.2 ± 3192.6	2.43 (0.022)	Group × Time	6.58 (0.002)
≥10,000 steps ^‡^	1st week	11,171.1 ± 1247.4	12,231.9 ± 2365.3	−1.55 (0.132)	Group	1.26 (0.271)
	7th week	11,778.7 ± 2062.3	10,877.5 ± 3026.5	0.95 (0.349)	Time	0.41 (0.665)
	12th week	12,788.9 ± 2275.1	10,373.4 ± 2164.2	2.91 (0.007)	Group × Time	8.59 (0.001)

Abbreviation: CG, control group; IG, intervention group; SD, standard deviation. ^†^ IG (*n* = 14), CG (*n* = 14); ^‡^ IG (*n* = 16), CG (*n* = 13).

**Table 4:** Effectiveness of a Mobile Wellness Program.

Characteristics		Pre M ± SD	Post M ± SD	Mean Differences between Time (Post–Pre)	t (*p*)
Sleep quality (PSQI total score)	IG	9.23 ± 3.18	7.50 ± 2.95	−1.70 ± 3.21	−1.53 (0.130)
	CG	8.73 ± 3.02	8.53 ± 2.82	−0.22 ± 4.04	
Subjective sleep quality	IG	1.63 ± 0.49	1.30 ± 0.46	−0.33 ± 0.60	−2.29 (0.025)
	CG	1.43 ± 0.62	1.50 ± 0.57	0.11 ± 0.84	
Sleep latency	IG	2.13 ± 0.86	1.76 ± 0.93	−0.36 ± 0.71	−0.77 (0.445)
	CG	2.13 ± 0.68	1.86 ± 0.86	−0.22 ± 0.69	
Sleep duration	IG	1.13 ± 1.10	0.90 ± 1.06	−0.23 ± 1.10	−0.83 (0.408)
	CG	1.13 ± 0.97	1.06 ± 0.90	0.00 ± 0.99	
Habitual sleep efficiency	IG	1.00 ± 1.23	1.06 ± 1.17	0.06 ± 1.14	−0.21 (0.834)
	CG	0.96 ± 1.18	1.23 ± 1.30	0.14 ± 1.74	
Sleep disturbance	IG	1.23 ± 0.50	0.96 ± 0.31	−0.26 ± 0.44	−2.00 (0.049)
	CG	1.23 ± 0.50	1.23 ± 0.43	0.00 ± 0.55	
Use of sleeping medication	IG	0.40 ± 1.67	0.13 ± 0.43	−0.26 ± 1.72	0.14 (0.886)
	CG	0.43 ± 1.69	0.13 ± 0.57	−0.33 ± 1.79	
Daytime dysfunction	IG	1.70 ± 0.74	1.36 ± 0.71	−0.30 ± 0.59	−2.04 (0.045)
	CG	1.40 ± 0.81	1.50 ± 0.77	0.07 ± 0.78	
Exercise self-efficacy	IG	2.74 ± 1.62	3.47 ± 1.91	0.80 ± 1.76	2.57 (0.012)
	CG	3.25 ± 1.82	2.90 ± 1.73	−0.35 ± 1.59	
Intrinsic motivation for	IG	3.26 ± 0.36	3.71 ± 0.44	0.45 ± 0.53	2.78 (0.007)
exercise	CG	3.38 ± 0.63	3.45 ± 0.48	0.06 ± 0.52	
Self-rated fatigue	IG	92.63 ± 14.61	87.37 ± 16.00	−4.50 ± 12.48	−1.06 (0.295)
	CG	94.83 ± 13.90	93.63 ± 19.00	−1.33 ± 9.82	
Wellness	IG	2.98 ± 0.47	3.42 ± 0.55	0.44 ± 0.35	4.14 (0.000)
	CG	3.17 ± 0.42	3.26 ± 0.51	0.06 ± 0.32	

Abbreviation: CG, control group; IG, intervention group; PSQI, Pittsburgh Sleep Quality Index; SD, standard deviation.

## Data Availability

The datasets generated and/or analyzed during the current study are not publicly available due to concerns regarding privacy, but select data are available from the corresponding author upon reasonable request.
